# The Use of Ultraviolet Irradiation to Improve the Efficacy of Acids That Are Generally Recognized as Safe for Disinfecting Fresh Produce in the Ready-to-Eat Stage

**DOI:** 10.3390/foods13111723

**Published:** 2024-05-31

**Authors:** Ruxianguli Maimaitiyiming, Yuting Yang, Ailikemu Mulati, Aihemaitijiang Aihaiti, Jiayi Wang

**Affiliations:** National Demonstration Center for Experimental Biology Education, Xinjiang Key Laboratory of Biological Resources and Genetic Engineering, College of Life Science and Technology, Xinjiang University, Urumqi 830046, China

**Keywords:** organic acids, ready-to-eat, UV-C, disinfection, lettuce, cherry tomato

## Abstract

Fresh-cut produce is usually produced under standardized disinfection processes, which are unavailable at the ready-to-eat stage. Currently, chemical sanitizers are used for washing, but their disinfection efficacy is limited. In this study, UV-C (1.03 kJ/m^2^) was combined with organic acids that are generally recognized as safe (GRAS), including citric, malic, acetic, and lactic acids (LAs), to wash lettuce and cherry tomatoes that are contaminated with *Escherichia coli* O157:H7 and *Salmonella* Typhimurium. The results showed that LA was the most effective treatment among the single treatments, with a pathogen reduction and cross-contamination incidence of 2.0–2.3 log CFU/g and 28–35%, respectively. After combining with UV-C, the disinfection efficacy and cross-contamination prevention capacity of the four GRAS acids significantly improved. Among the combination treatments, the highest pathogen reduction (2.5–2.7 log CFU/g) and the lowest cross-contamination incidence (11–15%) were achieved by LA-UV. The analyses of ascorbic acid, chlorophyll, lycopene, antioxidant capacity, and ΔE indicated that neither the single nor combination treatments negatively affected the quality properties. These results provide a potential hurdle technology for fresh produce safety improvement at the ready-to-eat stage.

## 1. Introduction

Fresh produce is an important source of daily nutrients, but contaminating pathogens seriously threaten consumer health. *Salmonella* and *Escherichia coli* O157:H7 account for 47.65% and 30.87%, respectively, of the infections resulting from the consumption of fresh produce [1]. Only recently, on 17 April 2024, *Salmonella*-contaminated fresh basil resulted in 12 illnesses and one hospitalization in seven states in the United States [2]. Therefore, sanitization is very important to ensure the safety of ready-to-eat fresh produce.

To increase the freshness characteristics of ready-to-eat produce, nonthermal processing technologies such as plasma, pulsed light, ultrasound, and ultraviolet have been widely studied [3]. However, the produce disinfection method in the ready-to-eat stage requires convenience [4]. Thus, chemical disinfectants are widely used. Among these disinfectants, chlorine-based disinfectant is widely used, as it has the advantages of moderate efficacy and a low working concentration (100–200 ppm) [5]; however, there is concern over the formation of carcinogenic and mutagenic byproducts (e.g., chloroform and trihalomethanes) during washing [6]. Most organic acids are approved as GRAS by the FDA and are used as food additives in the food industry. In this respect, organic acids are better than sodium hypochlorite [6]. Acetic (AA), malic (MA), lactic (LA), and citric acids (CAs) are commonly used to treat fresh-cut produce [7].

Finten et al. [8] found that 0.5% CA is superior to 200 ppm chlorine in controlling *Listeria monocytogenes* and *E. coli* growth on spinach leaves. LA washing reduces *L. innocua* by 1.0 log CFU/g on broccoli compared with chlorine washing [9]. However, the disinfection efficacy of each single treatment is limited, and combining different technologies to develop hurdle methods is an emerging trend. For example, Kang et al. [10] combined cinnamon leaf oil with LA to inactivate *L. monocytogenes* on fresh-cut Treviso and observed a reduction of 2.9 log CFU/g, significantly higher than that of single treatments and chlorine. The efficacy of MA against *E. coli* and *L. innocua* on fresh produce is significantly increased after combining it with pulsed light [11]. Although the sequential use of chemical disinfectants and other methods increases the disinfection efficacy, the increased inconvenience decreases the application potential in the ready-to-eat stage (e.g., families and restaurants). Therefore, a method that can work simultaneously with chemical washing is required to improve disinfection efficacy.

UV-C is a low-cost and easy-to-use disinfection method. The aerobic mesophilic bacteria present on fresh-cut kiwifruit are significantly inactivated (>1.0 log CFU/g) by treatment with UV-C [12]. UV-C and sanitizers can simultaneously disinfect produce in water [13] without increasing the process complexity. Therefore, in this study, organic acid washing was combined with UV-C irradiation (suspended above the washing tank) to disinfect produce in water. The disinfection efficacy and effects on quality were analyzed based on lettuce and cherry tomato models. 

## 2. Materials and Methods

### 2.1. Sample Preparation 

Lettuces and cherry tomatoes were purchased at a local supermarket and used within 3 days. The outer leaves, inner baby leaves, and stem of the lettuce were removed. The remaining part and the cherry tomatoes were rinsed under tap water for 30 s. The lettuce was cut into pieces (diameter of 5.2 cm) for further use. 

### 2.2. Inoculation

*E. coli* O157:H7 (NCTC12900) and *S.* Typhimurium (ATCC14028), recommended for fresh produce inoculation [14,15], were selected for this work. One colony of each pathogen was cultured in nutrient broth overnight at 37 ℃ and a shaking speed of 120 rpm. The bacterial precipitates were washed three times using 0.85% NaCl and resuspended to 10^9^ CFU/mL using 0.85% NaCl. Then, the lettuce and cherry tomatoes were mixed with a bacterial suspension at a ratio of 1:20 (*w*/*v*) and 1:2 (*w*/*v*), respectively [16]. After 2 min, the inoculated samples were transferred into a biosafety cabinet for air-drying. The samples were stored for 12 h at 4 °C to ensure the pathogen was sufficiently attached.

### 2.3. Wash Water Preparation

The sterilized homogenates of the lettuces and cherry tomatoes were vacuum filtered and mixed with tap water to prepare wash water with a COD value of 1415 ± 67 mg/L and 681 ± 84 mg/L for the lettuce and cherry tomatoes, respectively [13]. 

According to previous studies, the disinfection efficacy of organic acids did not significantly increase as the concentration increased from 0.5% to 2% [17,18]. Thus, the concentrations of AA, LA, CA, and MA were adjusted to 0.5% using the above wash water, with a pH value of 2.78, 2.34, 2.29, and 2.32, respectively. 

### 2.4. Disinfection

A system reported by Wang et al. [13] was used in this study. In brief, 8 L of wash water with the adjusted COD value was added into the washer to obtain a water depth of 5.3 cm. Fresh produce (800 g) was transferred into the washer. Then, the washer was transferred into a UV-C chamber (Shijiashike, Liaoyang, China) equipped with four UV-C lamps. The distance between the water surface and the UV-C lamp was 20 cm. When the UV-C dose was increased from 0 to 1.71 kJ/m^2^, the fastest inactivation rate of pathogens on leaf green was observed by Kim et al. [19]. The washing time in the ready-to-eat stage is recommended to be no more than 5 min [4,20]. Thus, in this work, the processing time was 3 min to obtain a UV dose of 1.03 kJ/m^2^. 

### 2.5. Microbiological Analysis

After washing, the sample was dewatered using a manual salad spinner. The sample (25 g) was transferred into a stomacher bag containing 225 mL of 0.85% NaCl for 2 min of homogenization. The serially diluted bacterial suspension (0.1 mL) was surface-plated onto modified sorbitol MacConkey agar (Hopebio, Qingdao, China) and xylose lysine deoxycholate agar (Hopebio) and incubated for 24 h at 37 °C to analyze the levels of *E. coli* O157:H7 and *S.* Typhimurium, respectively. The samples washed with tap water were selected as the control group. The results were expressed as the count reduction, which is defined as the difference in microbial counts between the control and treatment groups.

### 2.6. Cross-Contamination Incidence Analysis

The cross-contamination incidence was analyzed according to Wang et al. [21]. In brief, the two-pathogen suspension was poured into the washer containing the two produce types (not inoculated with pathogens) and washing water, as described in Section 2.4. The pathogen concentration in the washer was 10^5^–10^6^ CFU/mL. The washer containing tap water was the control. After washing, the inoculated counts in the treatment group were divided by those in the control group, and the obtained value was defined as the incidence of cross-contamination.

### 2.7. Color Analysis

Five leaves and five cherry tomatoes in each group were randomly selected for color analysis. The values of L*, a*, and b* were determined for three locations on each sample using a colorimeter (CR400; Konica Minolta, Osaka, Japan). The colorimeter was calibrated using a white standard plate (Y = 82.80, x = 0.3194, y = 0.3264) before every use. ΔE was selected to evaluate the overall color difference and was calculated using the following formula:

$$\Delta E=\sqrt{{\left(\Delta L^{*}\right)}^{2}+{\left(\Delta a^{*}\right)}^{2}+{\left(\Delta b^{*}\right)}^{2}},$$

where ΔL*, Δa*, and Δb* represent the differences between the treatment and the sample without any treatment.

### 2.8. Analysis of Nutrition Properties

After washing, five leaves of lettuce and five cherry tomatoes were ground using liquid nitrogen, and the contents of ascorbic acid, lycopene, chlorophyll, and antioxidant capacity were analyzed according to Wang et al. [13].

### 2.9. Statistical Analysis

Statistical analyses were performed using SPSS v.22 (SPSS Inc., Chicago, IL, USA). The data were compared using an analysis of variance, followed by Duncan’s multiple range tests (*p* < 0.05). Each experiment was independently performed three times. All data were expressed as the mean ± standard deviation.

## 3. Results

### 3.1. Effects of Different Treatments against Pathogens on Lettuce

**Figure 1:**
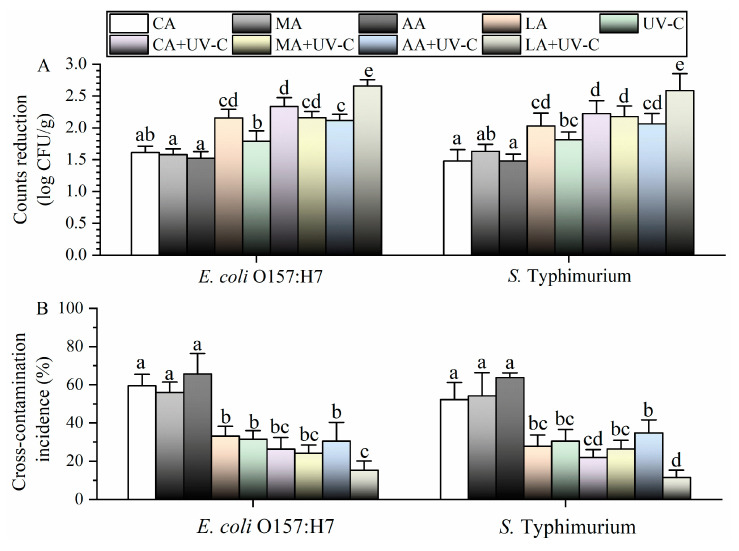
Disinfection efficacy (**A**) and cross-contamination control efficacy (**B**) of different treatments against pathogens on lettuce. Different lowercase letters indicate significant differences (*p* < 0.05).

*E. coli* O157:H7 on lettuce was inactivated by 1.5–1.6 log CFU/g using CA, MA, and AA (Figure 1A). After disinfection using LA and UV, *E. coli* O157:H7 was inactivated by 1.8 and 2.2 log CFU/g, respectively; these reductions were significantly greater than those for CA, MA, and AA. After combining CA, MA, and AA with UV, the observed count reductions (2.1–2.3 log CFU/g) were significantly greater than those for the single treatments. The highest inactivation efficacy (2.7 log CFU/g) was observed for LA-UV. Similarly, *S.* Typhimurium was inactivated by 1.8 and 2.0 log CFU/g when using UV and LA, respectively (Figure 1A); these reductions were significantly greater than those for CA, MA, and AA. After further combining with UV, the highest count reduction (2.6 log CFU/g) was achieved by LA-UV, and this reduction was significantly greater than those for the other treatments. 

The cross-contamination incidence of *E. coli* O157:H7 was 56–66% when employing CA, MA, and AA (Figure 1B); this incidence decreased to 31% and 33% when using UV and LA, respectively. The incidence was 24–30% after combining UV with CA, MA, and AA, which was significantly lower than that of the single treatments. The lowest incidence (15%) was observed using LA-UV. For *S.* Typhimurium, the incidence was 52–64% when using CA, MA, and AA and decreased to 28–30% when using UV and LA. The incidence was significantly decreased to 22–35% by CA-UV, MA-UV, and AA-UV, as compared with that for the single treatments. The lowest incidence (11%) of *S.* Typhimurium was also achieved by LA-UV.

### 3.2. Effects of Different Treatments against Pathogens on Cherry Tomatoes

**Figure 2:**
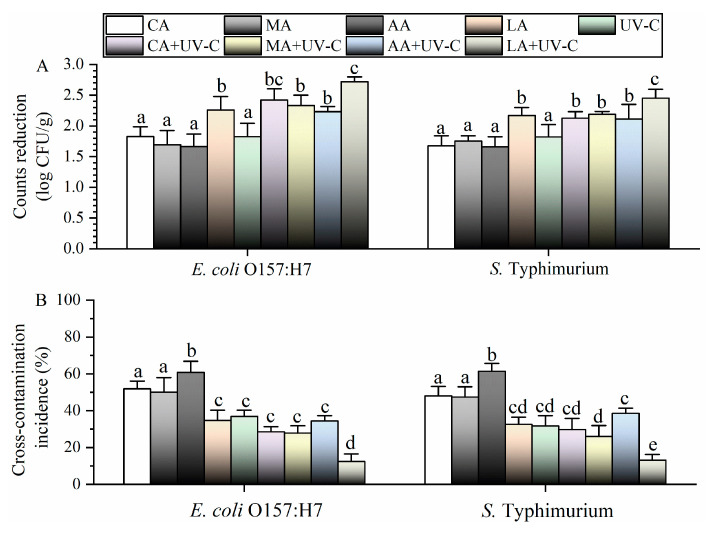
Disinfection efficacy (**A**) and cross-contamination control efficacy (**B**) of different treatments against pathogens on cherry tomatoes. Different lowercase letters indicate significant differences (*p* < 0.05).

When single treatments were used on cherry tomatoes, *E. coli* O157:H7 and *S.* Typhimurium were inactivated by 1.7–2.3 log CFU/g and 1.7–2.2 log CFU/g, respectively (Figure 2A). After combining with UV, the inactivation efficacy of AA, MA, and CA against *E. coli* O157:H7 and *S.* Typhimurium increased significantly to 2.2–2.4 log CFU/g and 2.1–2.2 log CFU/g, respectively. The highest count reductions for *E. coli* O157:H7 (2.7 log CFU/g) and *S.* Typhimurium (2.5 log CFU/g) were also achieved using LA-UV. After washing with CA-UV, MA-UV, and AA-UV, the cross-contamination incidence of *E. coli* O157:H7 and *S.* Typhimurium was 35–37% and 32–33%, respectively, which were significantly higher than those of CA, MA, and AA. When we combined LA with UV, the lowest cross-contamination incidence of *E. coli* O157:H7 (12%) and *S.* Typhimurium (13%) was observed.

### 3.3. Effects of Different Treatments on Quality Indicators in Lettuce and Cherry Tomatoes

**Figure 3:**
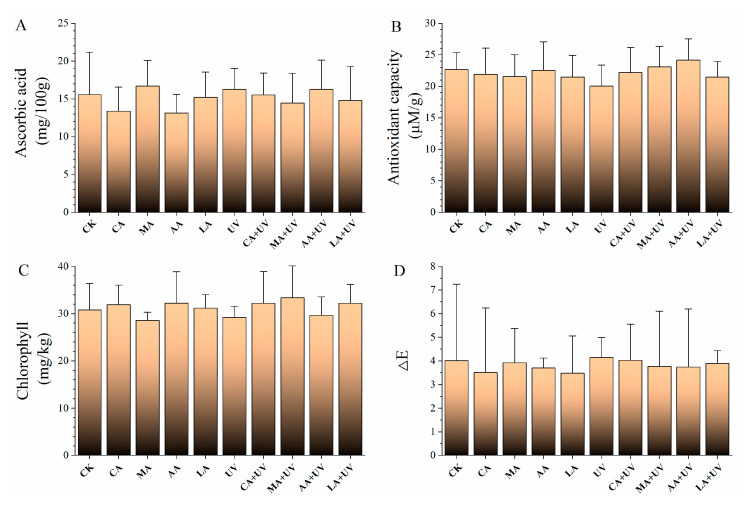
Effects of different treatments on the quality properties of lettuce. No significant difference was observed. (**A**–**D**) indicate ascorbic acid, antioxidant capacity, chlorophyll, and ΔE, respectively.

The content of ascorbic acid in lettuce was 15.6 mg/100 g (Figure 3A), and the content was not negatively affected after using acids and UV. The antioxidant capacity was also not affected as employing single nor combination treatments (Figure 3B). The color property analysis indicated that the chlorophyll content and ΔE in the control were 30.8 mg/kg and 4.0 (Figure 3C,D), respectively. After treatment with the single and combination treatments, these two properties were not significantly affected. 

**Figure 4:**
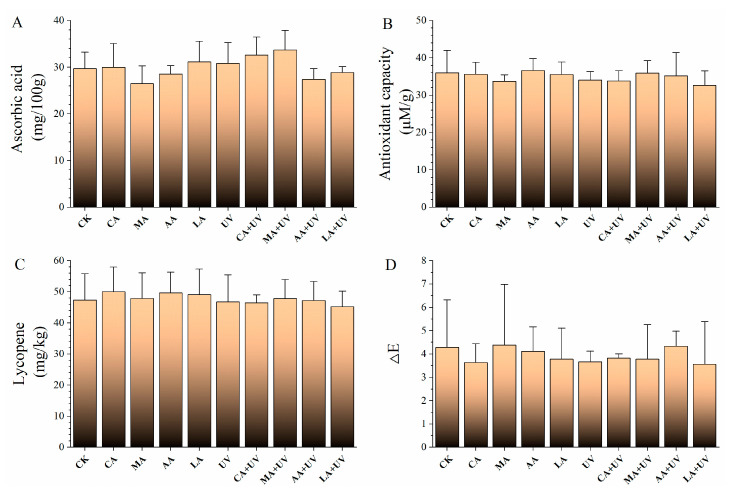
Effects of different treatments on the quality properties of cherry tomatoes. No significant difference was observed. (**A**–**D**) indicate ascorbic acid, antioxidant capacity, lycopene, and ΔE, respectively.

The ascorbic acid content and antioxidant capacity of cherry tomatoes were 29.7 mg/100 g and 36.0 μM/g (Figure 4A,B), respectively. After treatment with single and combination treatments, these two properties were not affected. Similarly, lycopene content was not affected after treatment with the single and combination treatments (Figure 4C). The color analysis showed that ΔE in the treatment groups was 3.6–4.3 (Figure 4D), which was not significantly different from that of the control group.

## 4. Discussion

The antibacterial activities of organic acids are attributed to the action of cellular anions [22]. The undissociated acidic anions penetrate the microbial cell membrane, and the higher intracellular pH promotes acid dissociation [23]. The dissociated anions exert toxic effects by damaging the membrane and inhibiting protein synthesis [24]. Among the four acids, the highest inactivation capacity achieved by LA was due to its relatively lower molecular weight (i.e., easier penetration), leading to the accumulation of intracellular lactate anions [25,26]. 

When two different disinfection techniques were combined, an additional count reduction was obtained compared to the single treatments. When combining ozone rinsing with ultrasound-assisted washing, the *E. coli* O157:H7 and *S.* Typhimurium present on lettuce were additionally inactivated by 0.5 and 0.6 log CFU/g, respectively, as compared with the single treatments [27]. After combining plasma with ultrasound, *E. coli* O157:H7 and *S.* Typhimurium on blueberries were additionally reduced by 0.9 and 0.7 log CFU/g, respectively, compared with ultrasound washing [28]. The aerobic bacteria, mold, and yeast present on lettuce can be inactivated by 2.3 and 1.3 log CFU/g by CA-H_2_O_2_, respectively, whereas the count reduction achieved by single treatments is less than 1.0 log CFU/g [29]. When UV was combined with organic acids in the present work, additional pathogen reduction was also observed. Similarly, a combination of cinnamon bark oil emulsion washing and UV achieved a 1.4 log CFU/g reduction in *S.* Typhimurium, and this reduction was significantly greater than those of the single treatments [30]. 

In the fresh-cut industry, washing water is circulated for use [31]. When pathogen-contaminated produce enters the water, pathogens on the produce’s surface enter the circulated water and contaminate the subsequent incoming produce, increasing the contamination incidence [32]. Therefore, the cross-contamination incidence control capacity of sanitizers should be evaluated in the fresh-cut industry. Although in the ready-to-eat stage (e.g., homes and restaurants), in most cases, the water is dumped after the produce is washed once [4], the cross-contamination incidence was also evaluated in the present work to make a comprehensive analysis. The results showed that LA was the most effective among the four acids in controlling cross-contamination, which is consistent with the results obtained by Van Haute et al. [31]. 

The content of ascorbic acid, lycopene, and chlorophyll and the antioxidant capacity are important nutrient properties in fresh produce. The total color difference (ΔE) is also one of the factors that determines the overall liking extent of consumers for produce. Previous studies have shown that the decolorizing capacity of the oxidizing agent was stronger than that of acids and UV. Bermúdez-Aguirre et al. [33] found that ΔE values were significantly increased after treatment with ozone, whereas they were not affected by treatment with CA and UV. Poimenidou et al. [34] found that the b* value of lettuce was 37.1 after washing with sodium hypochlorite, which is significantly higher than the value achieved by LA and AA. In this work, we selected organic acids as sanitizers and found that neither the single nor combination treatments negatively affected the nutrition and color properties of fresh produce. 

## 5. Conclusions

This study used four GRAS organic acids (LA, AA, MA, and CA) as washing sanitizers combined with UV to investigate their disinfection efficacy against lettuce and cherry tomatoes to reduce food-borne pathogen contamination in the ready-to-eat stage. The main conclusions were (1) the disinfection efficacy of LA against *E. coli* O157:H7 and *S.* Typhimurium was significantly higher than that of UV, MA, AA, and CA; (2) the disinfection efficacy and cross-contamination prevention capacity of the combination treatments were superior to those of LA, AA, MA, and CA; and (3) neither the single nor combination treatments led to a loss in nutrition and color properties. These results provide a reference for equipment construction for the washing step for fresh produce in the ready-to-eat stage. In future studies, different concentrations of organic acid could be combined with different dosages of UV to obtain a more effective disinfection system.

## Data Availability

The original contributions presented in the study are included in the article, further inquiries can be directed to the corresponding author.
